# Divergent responses of native and invasive macroalgae to submarine groundwater discharge

**DOI:** 10.1038/s41598-023-40854-7

**Published:** 2023-08-26

**Authors:** Angela Richards Donà, Celia M. Smith, Leah L. Bremer

**Affiliations:** 1 School of Life Sciences, University of Hawai‘i at Mānoa, Honolulu, HI USA; 2 University of Hawai‘i Economic Research Organization, University of Hawai‘i at Mānoa, Honolulu, HI USA; 3 Water Resources Research Center, University of Hawai‘i at Mānoa, Honolulu, HI USA

**Keywords:** Photosynthesis, Natural variation in plants

## Abstract

Marine macroalgae are important indicators of healthy nearshore groundwater dependent ecosystems (GDEs), which are emergent global conservation priorities. Submarine groundwater discharge (SGD) supports abundant native algal communities in GDEs via elevated but naturally derived nutrients. GDEs are threatened by anthropogenic nutrient inputs that pollute SGD above ambient levels, favoring invasive algae. Accordingly, this case study draws on the GDE conditions of Kona, Hawai‘i where we evaluated daily photosynthetic production and growth for two macroalgae; a culturally valued native (*Ulva lactuca*) and an invasive (*Hypnea musciformis*). Manipulative experiments—devised to address future land-use, climate change, and water-use scenarios for Kona—tested algal responses under a natural range of SGD nutrient and salinity levels. Our analyses demonstrate that photosynthesis and growth in *U. lactuca* are optimal in low-salinity, high-nutrient waters, whereas productivity for *H. musciformis* appears limited to higher salinities despite elevated nutrient subsidies. These findings suggest that reductions in SGD via climate change decreases in rainfall or increased water-use from the aquifer may relax physiological constraints on *H. musciformis*. Collectively, this study reveals divergent physiologies of a native and an invasive macroalga to SGD and highlights the importance of maintaining SGD quantity and quality to protect nearshore GDEs.

## Introduction

Across the tropical Pacific, native marine algae often have high cultural, ecological, nutritional, and economic value^1–3^ and can be important indicators of healthy nearshore groundwater dependent ecosystems (GDEs)^3, 4^. Submarine groundwater discharge (SGD) to intact coastlines of tropical high islands delivers a nearly ceaseless supply of nutrient-rich freshwater that sustains GDEs and their primary producers^5–7^. In relatively unperturbed watersheds, groundwater delivers naturally elevated levels of nutrients at a rate of discharge and salinity that varies with hydrological seasons and sea levels through tidal cycles^8^. The off-shore waters that surround these islands often have nutrient levels so low that dissolved inorganic nitrogen (e.g., nitrate as primary N type in SGD) and dissolved inorganic phosphate—essential macronutrients for growth by all plants—are undetectable by standard methods^5, 7^. In this context, natural SGD is a critical source of nutrients that has allowed for evolutionary diversity and moderate abundances of marine macroalgae within GDEs^4^. Although most macroalgae species do not tolerate freshwater^6, 9^, research from Hawaiʻi shows that some native species tolerate and even thrive in lower salinities characteristic of natural SGD plumes^6, 10, 11^. Anthropogenic inputs to natural SGD can add large loads of nutrient pollution to coastal ecosystems, while remaining generally unseen and infrequently measured^8^. Chronic imbalances resulting from these inputs increase biomass of invasive algae^12^ and when coupled with climate change impacts such as reduction of rainfall, may threaten important SGD-dependent communities.

The Kona coastline of West Hawaiʻi Island is a GDE hotspot with over 50 groundwater discharge points^13^ that sustain the local biota. Because of the potential stress on GDEs from increased urban development and excessive groundwater withdrawal, the Keauhou aquifer, which feeds into Kona GDEs, is a priority area for watershed conservation initiatives^14^. This area is also characterized by an abundance of cesspools^15^ and other On-Site Disposal Systems (OSDS) that currently contribute untreated wastewater to coasts via the SGD pathway^16^. These anthropogenic shifts in the quality of SDG and links to macroalgae are unexplored in the context of the Hawaiʻi State Water Code, which requires that water be managed as a public trust and includes protections for native species, i.e., *Ulva lactuca*, critical to cultural and ecological values embedded within GDEs^17, 18^.

Invasive micro- and macroalgal blooms are a top concern for the management and health of coastal ecosystems worldwide (e.g., ^19–23^). On tropical coral reefs, some invasive algae are known to overgrow sessile coral, attenuating over 90% of solar irradiance required for coral growth^24–27^. On unperturbed reefs, turf, crustose corallines, and macroalgae compete with coral for habitat but are kept in balance by herbivory and nutrient limitation^28, 29^. These natural balances are threatened globally from anthropogenic nutrient inputs^30^, herbivore overharvesting^31^, and climate change^32^. In GDEs, climate change perturbations and/or land-based nutrient pollution from agriculture and urban development^15^ can shift the quality of SGD and may create conditions where invasive macroalgae become dominant^12, 33–35^. In Hawai‘i, over a dozen species of macroalgae have been introduced, intentionally and accidentally, and several have become ecological dominants in native coastal habitats^22, 24, 25, 36^. Importantly, there is growing concern over the potential establishment of *H. musciformis* along the nearshore, SGD-fed coral reefs of Kona, as SGD conditions change.

Despite the cultural and ecological importance of native marine algae^1, 37^, and the persistent threat posed by invasive macroalgae in Hawai‘i^22, 38, 39^, there is a paucity of research on the growth and physiologies of these organisms to shifting SGD conditions, limiting informed land and water management decisions. To help fill this gap, we quantified the changes in photosynthesis and growth of a culturally and ecologically important native Hawaiian species, *Ulva lactuca*, as well as the highly invasive macroalgae *Hypnea musciformis*. These species were specifically selected to help inform land and water management decisions on the Kona coast of Hawaiʻi Island because the native is abundant whereas the invasive is not yet present in the Kona GDE.

*Ulva lactuca* Linnaeus (limu pālahalaha) is a common marine green algal species with wide global distribution^40^. Limu pālahalaha is a beloved native alga and is used extensively in Native Hawaiian cultural practices and food preparation^37, 40^. Limu pālahalaha’s ability to respond rapidly to increased nutrients allows it to thrive in natural, as well as degraded, high nutrient Hawaiian island habitats^40^. In contrast, *Hypnea musciformis* (Wulfen) Lamouroux is a weedy Atlantic species that was intentionally introduced to O‘ahu as a commercial carrageenan source^39^. Although extraction plans were quickly abandoned, *H. musciformis* rapidly spread and is now abundant—up to 80% cover in Mā‘alaea Bay, Maui agriculture and wastewater outputs—and in other nutrient-rich areas of Maui, O‘ahu, and Kaua‘i^8, 22, 39, 41^. This highly invasive species uses apical hooks to attach to other plants and substrate, fragments easily^22^, and forms large, floating, shallow-water mats that strand on beaches leading to substantial revenue losses^42^. It is also apparently absent in the Kona GDE habitats^41, 43^. Whereas native *U. lactuca* and invasive *H. musciformis* occupy some of the same bloom habitats^44^, it is unknown whether *H. musciformis* can co-exist in the full range of native habitat parameters where *U. lactuca* thrives.

The objective of this study was to probe the performance of these two algae under SGD conditions that simulated the salinity/nutrient gradient from oligotrophic ocean to naturally-occurring nutrient-rich groundwater in Kona^10^. For these, we used the ecological gradients defined in previous hydrologic modeling studies^10, 13, 15, 45^. To achieve this objective, we examined whether *Ulva lactuca* and *Hypnea musciformis* (hereafter referred to as *Ulva* and *Hypnea*) growth and photosynthesis respond differently under six salinity (‰) / nitrate (μM) / phosphate (μM) treatments (T0: 35 / 0.5 / 0.005, T1: 35 / 14.3 / 0.005, T2: 28 / 27 / 0.15, T2.5: 28 / 53 / 1.64, T3: 18 /53 / 1.64, T4: 11 / 80 / 3.8) at three temperatures (~ 20, 27, and 30 °C; Table 1). Specifically, we tested the hypothesis that *Hypnea* growth and photosynthesis would decrease as conditions became more similar to SGD discharge points (e.g., like T4), in contrast to the increase expected in *Ulva*. We further explored the role of saturated photosynthesis rates combined with quantitation of diurnal irradiance, at the individual plant level, to determine whether this integrated characterization of algal physiology could improve our ability to model algal growth. Such models would be useful tools for future eco-hydrology work, using algae as proxies for health and habitat studies, and would expand our ability to better understand current and forecast future disturbances and population shifts. The ability to forecast changes in marine ecosystems is particularly important considering the predicted effects of future land-use and climate change. Changes in SGD quantity and/or quality could significantly reduce physiological barriers to entry for any fully-marine invasive algae, particularly those with successful fragmentation and attachment strategies as found for this species of *Hypnea*.

**Table 1:** Salinity and nutrient values for all treatments during the experimental period. T1–4 were part of the initial experimental design. T0 and T2.5 (bold) were added to fill data gaps. All treatments were run at ~ 20, 27, or 30 °C. Runs 1, 2, 4 and 9 were double runs with several days of overlap. Runs indicate specific species only when not run together. *Run 9 *Ulva* (T2.5) removed from the dataset due to unrelated tissue sloughing.

Treatment #	**T0**	T1	T2	**T2.5**	T3	T4
Salinity (‰)	**35**	35	28	**28**	18	11
Nitrate (μmol)	**0.5**	14.3	27.1	**52.9**	52.9	80.0
Phosphate (μmol)	**0.005**	0.005	0.15	**1.64**	1.64	3.79
Runs	**6, 6.5 (Ul)** **8, 9 (Hm)**	1, 2, 3, 3.5, 4	1, 2, 3, 3.5, 4	**7 (Hm)** 9 (Ul)*	1, 2, 3, 3.5, 4	1, 2, 3, 3.5, 4

## Results

This investigation generated data via replicated in vitro assessments of photosynthesis and growth of two contrasting species over nine day runs, for 16 replicates each across three water temperatures (~ 20, 27, 30 °C) and six combinations of salinity and nutrients (T0–T4; See above or Table 1). We documented daily ambient irradiances during each run across several seasons.

The variance explained by the linear mixed-effect models (LMM) for all dependent variables analyzed (maximum photosynthesis, P_max_; irradiance saturation, E_k_; diurnal saturation time, H_sat_; diurnal saturated photosynthesis index, DSPI; rel-H_sat_ [data in Supplemental material]; 9-day growth), increased with the addition of the chosen random effects, emphasizing the importance of inherent plant variability (plant ID), irradiance changes over time (run), and other unmeasured variables. These data are reported in R^2^m and R^2^c columns in Table 2. Overall, the LMM was a better fit for *Ulva* than for *Hypnea* for fixed effects (treatment and temperature), explaining up to 62% of the variance in *Ulva* and 38% in *Hypnea*. Both of these highest values were from the E_k_ model fit. With the addition of random effects, the explained variance increased to a maximum 86% in *Ulva* and 87% in *Hypnea—*both from the DSPI model fit. In *Ulva*, treatment had a significant effect on all dependent variables tested, whereas temperature only affected the outcome in one analysis (E_k_). The significant results were more complex in *Hypnea* as treatment was an important factor affecting the outcomes in four of the six dependent variables, but temperature affected outcomes in all but one (growth). All significant test results, model coefficients of determination, and random effects used in each analysis are provided in Table 2.

**Table 2:** Statistical output for linear mixed-effects model (LMM) fit providing marginal and conditional R^2^ values. P-values, chi squared (χ^2^), and degrees of freedom (df) obtained from likelihood ratio tests for effects of treatment and temperature. Data are divided by species. Total number of observations for each analysis: *Ulva* = 240, *Hypnea* = 286. Random effects in parentheses below R^2^c (a = plant ID, b = run, c = RLC order, d = lunar phase).

	*Ulva*						*Hypnea*					
	Likelihood ratio tests				LMM fit		Likelihood ratio tests				LMM fit	
	Treatment		Temperature				Treatment		Temperature			
	P	χ^2^ (df)	p	χ^2^ (df)	R^2^m	R^2^c	p	χ^2^ (df)	p	χ^2^(df)	R^2^m	R^2^c
P_max_	< 0.001	130.1(4)	0.151	3.8(2)	0.59	0.69 (abc)	0.008	15.8(5)	< 0.001	40.1(2)	0.27	0.63 (abc)
E_k_	< 0.001	140.7(4)	0.033	6.8(2)	0.62	0.72 (abc)	0.056	10.8(5)	< 0.001	54.5(2)	0.38	0.57 (ab)
H_sat_	< 0.001	91.3(4)	0.363	2.0(2)	0.38	0.85 (ab)	< 0.001	23.4(5)	0.001	13.6(2)	0.19	0.74 (ab)
DSPI	0.007	14.0(4)	0.517	1.3(2)	0.14	0.86 (abc)	0.171	7.8(5)	0.004	11.1(2)	0.19	0.89 (ab)
Growth	< 0.001	69.9(4)	0.952	0.1(2)	0.26	0.73 (ad)	< 0.001	49.8(5)	0.282	2.5(2)	0.15	0.51 (abc)

Both *Ulva* and *Hypnea* exhibited a consistently larger difference in means between T0 and T1 than between any other adjacent treatment pairs along the gradient—for most dependent variables. This highlights the large nutrient increase (+ 2700% nitrate) from T0 (oligotrophic) to T1 (oceanic salinity + elevated nutrients) but may also reflect some reduced variability in individual plants. T0 plants were all collected in February (*Ulva*) and October (*Hypnea*) 2022, and *Hypnea* T2.5 individuals were collected in April 2022, whereas T1–4 were made up of a combination of plants from time periods with a broader range of diurnal irradiance. Plant variability was accounted for in the model’s random effects and trends remained consistent even when T0 was removed from the analysis. Accordingly, the results are herein presented primarily as changes in elevated nutrient treatments T1–T4 versus oligotrophic T0 because this is the most relevant comparison for our study.

### Maximum photosynthetic rate (P_max_)

**Figure 1:**
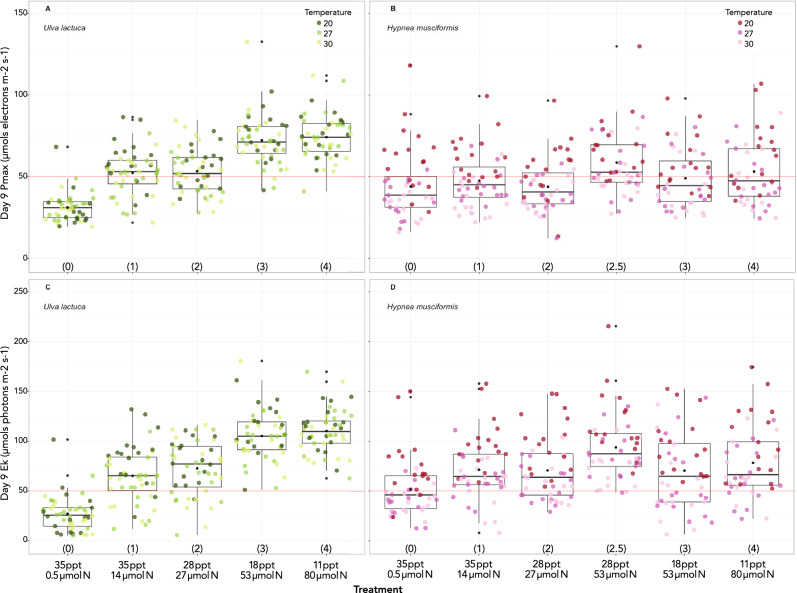
Day 9 P_max_ (**A**, **B**) and E_k_ (**C**, **D**) by treatment for *Ulva* (green points) and *Hypnea *(red points). Black dots denote means for each treatment. Treatment description and number (in parentheses) on x-axis. Shade of colored dot represents temperature. Red lines mark 50, an arbitrary value for comparison.

P_max_ is a value that describes the maximum irradiance-dependent photosynthetic rate. In *Ulva*, P_max_ linearly increased and more than doubled from T0 to T4. The treatment with the highest nutrient input and lowest salinity (4) also had the highest measured P_max_ at 74.3 μmols electrons m^−2^ s^−1^. Because T0 was the baseline oceanic salinity/ nutrient treatment, we found that all Ts 1–4 affected P_max_ (χ^2^ (4) = 130.1, p < 0.001), increasing it by a maximum of 43.5 (± 3.5 std. err.) μmols electrons m^−2^ s^−1^ (Fig. 1A; Table 3). Temperature did not have a significant effect on P_max_ (χ^2^ (2) = 3.8, p = 0.151).

**Table 3:** Mean values for each species and treatment for D9 P_max_ (μmols electrons m^−2^ s^−1^) and E_k_ (μmols photons m^−2^ s^−1^), H_sat_ time in minutes, DSPI (mol m^−2^), and 9-day growth (%). *Ulva T2.5 experienced excessive tissue sloughing as a likely reproductive response to lunar phase and was not included here.

Treatment	Species	P_max_	E_k_	H_sat_ time	DSPI	Growth
35‰/0.5 μmol N (T0)	Ul	31.1	27.2	507	3.4	4.5
	Hm	43.9	51.8	469	3.5	18.2
35‰/14 μmol N (T1)	Ul	52.3	65.2	404	4.4	30.6
	Hm	47.5	71.4	414	4.4	35.3
28‰/27 μmol N (T2)	Ul	53.3	72.8	398	4.5	38.1
	Hm	43.8	70.7	421	4.2	19.9
28‰/53 μmol N (T2.5)	Ul*	NA	NA	NA	NA	NA
	Hm	58.7	93.8	410	5.2	34.6
18‰/53 μmol N (T3)	Ul	72.3	105.3	344	4.6	45.4
	Hm	49.0	70.5	420	4.3	23.8
11‰/80 μmol N (T4)	Ul	74.3	110.4	333	4.6	50.0
	Hm	53.1	78.3	394	4.4	11.0

Treatment affected P_max_ in *Hypnea* (χ^2^ (5) = 15.8, p = 0.008) increasing it by a maximum of 14.7 μmols electrons m^−2^ s^−1^ (± 11.5 std. err.) in T2.5. The effect of temperature was also significant (χ^2^ (2) = 40.1, p < 0.001) decreasing P_max_ by 17.3 (± 3.1 std. err.) at 27 °C and by 19.4 (± 2.7 std. err.) at 30 °C (Fig. 1B). Notably, 27 °C is close to ambient seawater temperature.

### Irradiance saturation (E_k_)

E_k_ denotes the irradiance at which an individual transitions from irradiance-limited to substrate-limited photosynthetic performance. E_k_ ranged from 27.2 (T0) to 110.4 (T4) μmols photons m^−2^ s^−1^ in *Ulva* indicating a strong, increasing relationship with increasing SGD conditions (Fig. 1C). Salinity/nutrient treatments affected E_k_, (χ^2^ (4) = 140.7, p < 0.001) increasing E_k_ by up to 84.3 (± 7.2 std. err.), in T4. Temperature also affected *Ulva* (χ^2^ (2) = 6.8, p = 0.033), decreasing E_k_ by 10.5 ± 4.8 std. err. (at 27 °C) and 10.6 ± 4.4 std. err. (30 °C) μmols photons m^−2^ s^−1^.

E_k_ in *Hypnea* ranged from 51.8 (T0) to 93.8 (T2.5). Similar to P_max_ in *Hypnea*, E_k_ was affected by temperature (χ^2^ (2) = 54.5, p < 0.001) but not by treatment (χ^2^ (5) = 10.8, p = 0.056; Fig. 1D). E_k_ decreased by 35.8 (± 4.2 std. err.) and 39.4 (± 4.2 std. err.) μmols photons m^−2^ s^−1^ at 27 and 30 °C, respectively, relative to 20 °C. E_k_ was the only dependent variable that was significantly affected by temperature in *Ulva*, whereas temperature was almost always a significant factor affecting *Hypnea*.

### Diurnal saturation time (H_sat_)

**Figure 2:**
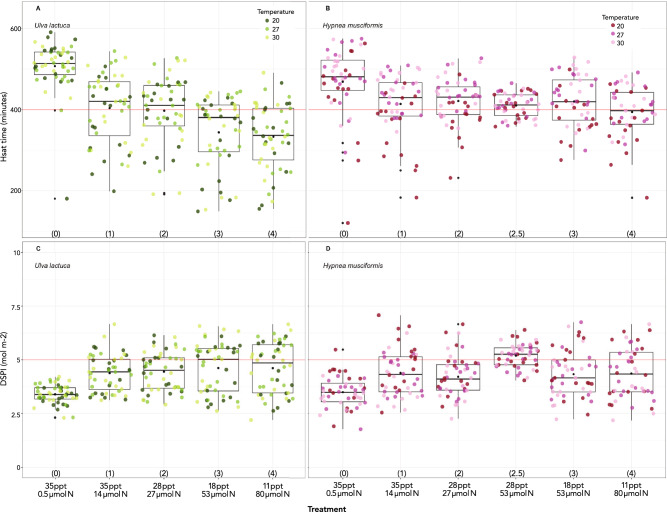
Saturation time in minutes (H_sat_; **A**, **B**) and DSPI in mol m^−2^ (**C**, **D**) for all treatments in *Ulva* (**A**, **C**) and *Hypnea* (**B**, **D**). Black dots denote means for each treatment. Treatment description and number (in parentheses) as in Fig. 1.

Diurnal saturation time describes the mean time (in minutes) plants spent in irradiance ≥ E_k_. The mean diurnal saturation time for *Ulva* treatments increased from 333 (T4) to 507 (T0) minutes. H_sat_ was most affected by the salinity/nutrient combination in T4 (χ^2^ (4) = 91.3, p < 0.001; Fig. 2A), which lowered it 183 min (± 50 std. err.) relative to T0. Temperature did not have a significant effect on *Ulva* H_sat_ (χ^2^ (2) = 2.0, p = 0.363).

The range for mean H_sat_ in *Hypnea* treatments was narrower than in *Ulva* and spanned 394 to 469 min (Table 3). Both treatment (χ^2^ (5) = 23.4, p < 0.001; Fig. 2B) and temperature (χ^2^ (2) = 13.6, p = 0.001) affected H_sat_, increasing it by a maximum of 41 min (± 11 std. err.) at 27 °C relative to 20 °C individuals.

### Natural irradiance changes during experimental period

**Figure 3:**
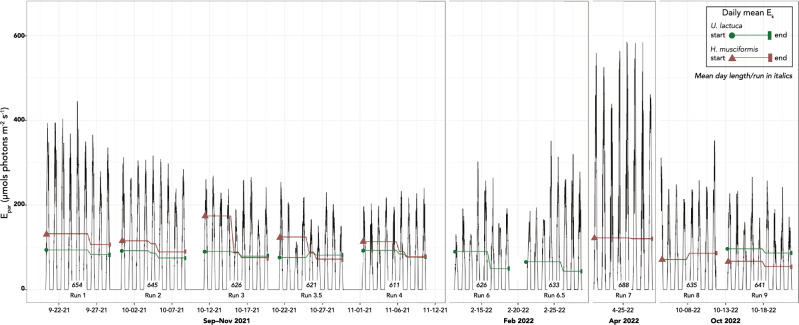
Irradiance during experimental runs by day. Green lines (*Ulva*) and red lines (*Hypnea*) mark daily mean E_k_ for all treatments from beginning of experiment to end. Mean E_k_ values were grouped by period as described in methods. Mean day length in minutes (total irradiance ≥ 1 μmols photons m^−2^ s^−1^) in italics for each run. Data in run 9 for *Ulva* were removed from the dataset due to lunar phase reproductive tissue sloughing unrelated to the experiment.

The initial experimental period ranged from September to early November 2021. The mean day length for September 20–29 (run1) was 654 min and decreased to 611 min by run4. Because we did not use artificial light, the requirement to conduct subsequent experiments on days of similar duration was imposed, thus mid-year months were avoided. In February and October 2022, mean day length ranged from 626 to 641 min. The day length maximum in April 2022 was 688 min—more than 30 min longer than the September 2021 maximum. This time differential exposed a potential inconsistency that was addressed by normalizing H_sat_ data relative to the total minutes available for photosynthesis (≥ 1 μmols photons m^−2^ s^−1^; rel-H_sat_; see supplemental materials). Daily irradiance for each run is shown with total day length in minutes (Fig. 3). Mean E_k_ values for both species show the general direction of change and are based on D1 and D5 measurements.

### Diurnal saturated photosynthesis index (DSPI)

The diurnal saturated photosynthesis index represents a simplified irradiance-only component of gross photosynthetic production and combines the maximal photosynthetic rate (P_max_) multiplied by the time in saturated irradiance (H_sat_), iterated every three days (or fraction thereof) for the 9-day experiment. Our results show that only treatment affected DSPI (χ^2^ (4) = 14.0, p = 0.007) in *Ulva,* increasing DSPI by 1.1 mol m^−2^ (± 0.8 std. err.) in T4 relative to the baseline T0 (Fig. 2C). Temperature did not significantly affect DSPI (χ^2^ (2) = 1.3, p = 0.517). The inverse occurred in *Hypnea* as only temperature affected DSPI (χ^2^ (2) = 11.1, p = 0.004) decreasing it by 0.3 mol m^−2^ (± 0.1 std. err.) in both 27 and 30 °C (Fig. 2D). Treatment had no effect (χ^2^ (5) = 7.8, p = 0.171) on *Hypnea* DSPI.

### Algal growth

**Figure 4:**
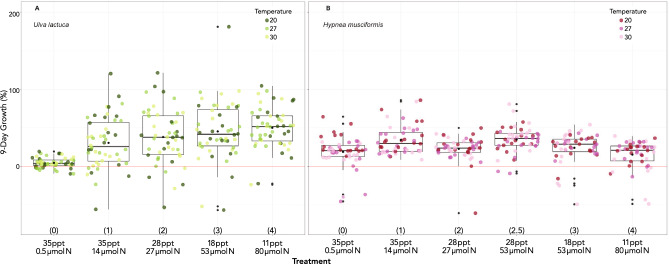
9-day growth (%) for *Ulva* (**A**) and *Hypnea* (**B**). All notations as in Fig. 1.

To determine whether photosynthesis correlates well with growth in these species, we first modeled growth as a function of salinity/nutrient combinations and temperature. As was the case in all other photosynthesis variables for *Ulva*, treatment affected growth (χ^2^ (4) = 69.9, p < 0.001), strongly increasing the 9-day growth to a maximum of 49.3% (± 6.3 std. err.) in T4 relative to baseline T0 (Fig. 4). Again, the effect of temperature was not significant (χ^2^ (2) = 0.1, p = 0.952). For *Hypnea*, treatment affected growth (χ^2^ (5) = 49.8, p < 0.001) but temperature did not (χ^2^ (2) = 2.5, p = 0.282). Nine-day growth in treatments 1 and 2.5 was virtually the same and increased by 15.3% (± 4.1 std. err.) and 16.0% (± 4.5 std. err.), respectively, relative to the baseline T0.

We then ran linear regressions with growth as the dependent variable and P_max_, E_k_, H_sat_, and DSPI as predictors. These showed that nearly all affected growth in *Ulva* (p-values for predictors in order: < 0.001*, < 0.001*, 0.290, < 0.001*). The photosynthesis variables were less effective for *Hypnea* (p-values in order: 0.038*, 0.091, 0.076, < 0.001*).

### DSPI as a predictor of growth

**Figure 5:**
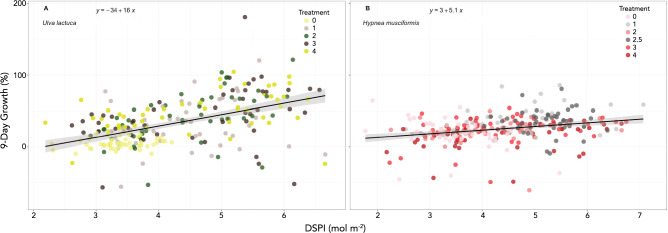
Linear regression for growth predicted by DSPI in (**A**) *Ulva* and (**B**) *Hypnea.* Colored dots represent treatments.

To better understand the relationship between DSPI and growth, we used an LMM adding treatment as a random effect and likelihood ratio tests as described in methods. DSPI was significantly correlated with growth in *Ulva* (χ^2^ (1) = 36.6, p < 0.001) increasing it 12.2% (± 1.9 std. err) and in *Hypnea* (χ^2^ (1) = 13.6, p < 0.001), increasing it by 4.1% (± 1.1 std. err; Fig. 5). R^2^c for combined fixed and random effects was 0.26 for *Ulva* and 0.15 for *Hypnea,* indicating potential species-specific factors for growth that remain unidentified (e.g., apical cell growth vs. diffuse cell division)*.*

## Discussion

Few physiological studies, to date, have examined native or invasive algae production as a function of habitat parameters in submarine groundwater discharge (SGD)-influenced groundwater dependent ecosystems (GDEs) that are common in oceanic high islands across the central Pacific region^5–8^. The Hawaiian native *Ulva lactuca* and invasive seaweed *Hypnea musciformis* were selected because they have contrasting ecologies and possess different evolutionary histories, yet their physiology and growth parameters in SGD conditions remain understudied.

Our in-depth investigation of photosynthetic parameters of these important algae details several facets of algal physiology, and highlights informative known and new parameters, i.e., E_k_, H_sat_, and DSPI, to yield an intuitively satisfying assessment for the first time. We further integrate the use of the irradiance saturation value, the value of photosynthetically active radiation (PAR) needed to attain steady state rates of photosynthesis, also known as E_k_^46–48^, and of maximal rates of photosynthesis coupled with irradiance, to estimate overall production in response to SGD conditions. Calculating the amount of time that a plant is at or above E_k_ irradiance during the experiments gives the novel ability to determine lengths of time individuals experienced steady state rates of maximal photosynthesis each day (H_sat_^49^). Because natural irradiance fluctuated throughout the experimental periods, and importantly, T0 and T2.5 were added subsequent to the initial experimental runs, we calculated H_sat_ relative to the day length (rel-H_sat_) to account for potential differences (see supplementary materials).

Following Zimmerman et al. (1994)^49^ oxygen electrode-based work with the temperate seagrass *Zostera marina*, we multiplied H_sat_ and P_max_ to derive the product, a novel diurnal saturated photosynthetic index, DSPI, as a new parameter using fluorescence-based PAM measurements to estimate daily maximum photosynthetic productivity in each individual. DSPI, thus represents a time-integrated maximal photosynthesis value that expands our ability to compare algal physiologies. Further, our results show that DSPI and growth are significantly correlated but DSPI falls short in explaining the high variability in *Ulva* and *Hypnea* growth. One explanation for the high variability in *Hypnea* growth may be its allocation of energy to survival and maintenance rather than growth when in new and nutrient-rich habitats^38^. Overall, these results highlight the need to better understand energy allocation in usually nutrient-limited marine algae so we may adequately assess the risks of nutrient increases to important ecosystems such as GDEs.

*Ulva* growth increased as treatments became more similar to SGD conditions near discharge points. The opposite relationship was found for *Hypnea*. The response dynamics were markedly different between species, however, because growth increased consistently for *Ulva* with each step towards hyposalinity, whereas *Hypnea* growth appeared to reach a maximum at 28‰. With an increase from 27 to 53 μmols N at 28‰, *Hypnea* was able to increase growth, but at the same nutrient level and a decrease in salinity to 18‰, *Hypnea* growth stalled and further declined as treatments extended further into hyposalinity/elevated nutrient scenarios. These divergent physiological responses allow us to better understand some of the mechanisms behind broad distributional patterns that *Ulva* exhibits in the field and emphasizes the strong marine environmental conditions under which invasive *Hypnea* blooms^40, 44^.

The LMM predicting growth as a function of DSPI showed that the relationship was significant, but the model fit was not as strong as expected, revealing that some irradiance and plant variabilities remain unexplained. Although they added to the variability in the response, irradiance changes experienced during the months-long experiment emerged as an unexpected positive attribute of the experimental design. In this manner, we were able to run the experiments over an extensive period to increase replication while also capturing the irradiance changes and distributing their effects across the treatments and temperatures. Furthermore, we did not address non-photochemical quenching in this study but recognize its potential to broaden our understanding of these photosynthetic physiologies^50^.

In summary, *Ulva* T4 individuals in 20 °C, representing the strongest, natural SGD conditions (lowest salinity, highest nutrients), had the highest rates of P_max_, the highest E_k_, and they spent the least time in H_sat_ (even when adjusted to rel-H_sat_%; see supplemental information). They also showed the highest DSPI of all treatments. Because growth was also highest in T4, these photosynthetic parameters appear to be optimized for life near SGD discharge points along basaltic coastlines.

*Hypnea* underperformed with decreasing salinity regardless of nutrient inputs, suggesting an intolerance to salinity 18‰ and below. The gap between 28‰ and 18‰ is wide open for future studies to narrow down and better define the salinity range that supports *Hypnea*, assuming an adequate nutrient subsidy. Temperature affected all photosynthetic parameters. P_max_, E_k_, and DSPI were highest and H_sat_ was lowest at 20 °C relative to 27 and 30 °C, which were highly similar. These temperature results likely reflect the optimization of this species to sub-tropical Florida before its introduction to Hawai‘i^22^ but also suggests that *Hypnea* could survive in SGD-types of temperatures in reef regions where salinities are moderately decreased but nutrients are elevated. Based on climate change predictions of reduced rainfall, such “sweet spots” are likely to occur because decreases in rain volume will increase salinity, whereas elevated nutrient inputs are likely to continue. These combined may create an SGD output that favors *Hypnea*’s physiology.

GDEs that are fed primarily by natural SGD sources, such as in coastal Kona, are fundamentally different from those on other islands in the Hawaiian archipelago because many of these receive large, anthropogenically-derived inputs that mix with the natural flows^6, 13^. The Kona coast is well known for high volumes of SGD without stream or overland inputs^13, 45^; the numerous, large, point-sourced SGD plumes and smaller diffuse flows discharge nutrient-rich fresh water as the only source of new nutrients for the native plant community^13^. Natural SGD discharge points along the Kona coastline support dense populations of *Ulva,* whereas *Hypnea* is apparently absent from the area. By contrast, abundant *Hypnea* biomass is found on other islands where excess nutrient loads from agriculture and/or wastewater feed into SGD flows^8, 38, 51^. Thus, changes that may impact the quality and quantity of SGD in Kona, are cause for concern. Current stressors, i.e., increasing OSDS densities, together with predicted increases in urban development and groundwater withdrawal, have made Kona’s Keauhou aquifer a priority for watershed conservation initiatives^14^. And although Hawai‘i law mandates that cesspools must be upgraded by the year 2050, water quality improvements have been slow and spatially variable^15^. In addition, centralized wastewater treatment plants that dispose of treated effluent in shallow coastal waters remain a threat to GDE health and may be in violation of the U.S. Clean Water Act. Experiments that test wastewater enrichment scenarios are underway but were not included in this study.

Climate change is expected to deliver less rain to already dry areas of the Hawaiian Islands, leading to decreasing SGD inputs to coastal regions^52^. Our results show that *Hypnea* is not well-suited to the current conditions of Kona coastal SGD habitats, but *Ulva* thrives in low salinity, high nutrient SGD ecosystems. We thus anticipate that with reduced freshwater input, coastal salinity will rise, increasing the potential for *Hypnea* to successfully establish in Kona. Predicted nutrient pollution of SGD will further enhance that likelihood. Localized anthropogenic eutrophication, persistent invasive macroalgal and phytoplankton blooms, as well as decreased reef biodiversity are features of these new GDE seascapes^8, 10, 12, 44, 53^. Once an invasive species is established in a new area, it can be extremely difficult to eradicate^20, 54, 55^ and can increase competitive interactions among invasives and natives in their now-changed ecosystem. Our study focused on the Kona coast SGD ecosystem, but similar conditions and vulnerabilities are found globally^56^. Importantly, each island, its SGD, and the composition of its biological community, may represent a continuum of SGD-biological species interactions^7^. Clearly, much more research is needed to better provide adaptive management of water resources that protect native species and minimize the establishment of aggressive invasives.

These results are also important in the context of subsistence collecting of limu pālahalaha (*Ulva*), and possibly other native limu, i.e., limu manauea (*Gracilaria coronopifolia*) and limu huluhuluwaena (*Grateloupia filicina*), that thrive and may have evolved under SGD conditions^41^. Protection of these organisms for the public trust is mandated by law, increasing the importance of protecting SGD quality and quantity to ensure native traditional practices are preserved. Wise stewardship of GDEs and their recharge would require improved land and water management decisions including more permeable urban surfaces, native ecosystem protection, and sustainable groundwater withdrawal rates, but would help to ensure groundwater dependent native communities are protected, to the benefit of all.

## Materials and methods

### Kona SGD coastline habitat

*Ulva* commonly co-occurs in persistent coastal blooms with the highly branched, cylindrical rhodophyte *Hypnea,* which is now found at numerous sites around the Main Hawaiian Islands, especially in Maui coastal waters^12, 44^. In Hawaiian ecosystems, both species are readily grazed—exhibiting similar top-down vulnerabilities^57^—and both have similar uptake rates of limiting macronutrients^44^. Strikingly, *Hypnea* has not been found in the intertidal region of the Kona coast^22^, despite numerous vectors for delivery. We calibrated our experimental treatment parameters to the natural intertidal SGD conditions at Kona, Hawai‘i to test the physiological responses of these two species to a range of salinity/nutrient combinations following a gradient from full oceanic to groundwater seep locations.

### Algal manipulative experiments

Eight experimental runs were conducted from September to November 2021 to arrive at 16 total replicates per treatment and temperature of the two co-occurring algal species. Each run began with the collection of six individual plants of *Hypnea* and *Ulva* in the intertidal zone during a minus tide at Wāwāmalu Beach and Ka‘alawai, O‘ahu. Plants and seawater were stored in a cooler and transported to the Marine Macrophyte Lab greenhouse at UH-Mānoa to acclimate and consume tissue nutrients for eight days before beginning the experiment. Acclimating plants were kept in approximately 3 L of unfiltered seawater in covered and aerated 10 L aquaria in a partially shaded area of the lab outdoor terrace. Tanks were divided in half with white plastic egg crate to keep plants—one of each species—separate, and seawater was not changed during this drawdown phase. Plant placement within tanks was rotated every two days to encourage homogeneous acclimations by both species.

Plants were sectioned into four replicate pieces—each 0.28–0.3 g in wet weight—and returned to the tanks with the extra plant biomass removed. The experimental run commenced the following morning at 08:00 with photographs, rapid light curves (RLCs), and wet weighing for each individual (24 per species). RLCs were run on randomly chosen replicates with a Junior PAM-III using WinControl version 3.29 software (Walz, GmbH, Germany). Optimal settings were pre-determined for concurrent use with both species: Actinic light length was 0:30, intensity values (E_par_) were set at 0, 65, 90, 125, 190, 285, 420, 625, and 820 μmols photons m^−2^ s^−1^, and the measuring light intensity was set to 8. F_t_ was offset to fluctuate around zero in seawater before beginning RLCs and probe location was chosen when the F_t_ value was in the normal range for each species and/or was in a consistent range at a minimum of three locations on the replicate. Each RLC took ~ 5 min and all 48 samples were completed before noon to avoid mid-day depressions in photosynthetic performance. These time constraints precluded multiple measurements on individuals.

After each RLC, replicates were carefully dried with paper towel three times and weighed with a Sartorius TE214S digital analytical balance (precision to 0.0001 g; Sartorius AG, Göttingen, Germany) then placed in 700 mL of treatment seawater in an individual 0.946 L (1 qt) glass jar per plant after Dailer et al. (2012)^51^. Seawater was pre-filtered with a 0.22 μm Millepore Stericup® filtration system (Millepore Sigma, Darmstadt, Germany), diluted with deionized water to reach the treatment salinity dilutions, and stored in darkened 20 L carboys. After all replicates were placed in clear-lidded jars, each received the appropriate addition of nitrate and phosphate (Table 1) and were fitted with air tubes that slightly agitated and aerated the water. Eight jars were nested in clear, plastic, water-filled bins, and were raised on white plastic egg crate above a 500-W titanium aquarium heater (Finnex TH-05005; Illinois, USA). Three daytime temperatures (~ 20, 27, and 30 °C) were maintained within the bins from 06:00 to 18:30 every day during the experiment. Heaters were scheduled to shut off at 18:30 to allow evening and nighttime temperatures to drop to ambient (~ 17–20 °C) in the air-conditioned greenhouse. Bins were aligned under a PVC pipe structure covered in shade cloth that reduced ambient light by ~ 50% with minimal obstruction.

Six treatments of paired salinity and nutrient concentrations simulated a gradient from coastal ocean to coastal spring conditions with decreasing salinity ranging from 35–11 ppt and increasing macronutrient ranging from 14–80 μmol nitrate and 0.005 to 3.79 μmol phosphate (T0–4; Table 1). Water changes and fresh inoculations were done every two days before noon and to avoid bin effects, each set of jars was moved counterclockwise to the adjacent bin and their positions within the bins were shifted from front to back. Temperatures were adjusted accordingly as described below. Wet weights were measured on Day 1 (D1), Day 5 (D5) and on Day 9 (D9), which concluded the experimental run and yielded a total of two replicates for each treatment, temperature, and species combination. The entire process was repeated seven more times with a final total of 16 replicates. Data gaps for both species were later identified, and each species underwent additional treatments to fill those gaps. The first added treatment (T0) was needed to test the nutrient-enriched treatments against oligotrophic oceanic conditions. The second added treatment combined the salinity of treatment T2 and the nutrients of treatment T3 to test a refinement of our hypothesis that increasing SGD was the primary cause of decrease in growth and photosynthesis in *Hypnea*. In these later iterations, all steps were repeated except the replication procedure. A total of 48 plants of each species were collected, acclimated, and one 0.28–0.3 g piece per plant was placed in 700 mL of seawater to commence the experiment, as above. At the end of the 9-day run, the number of replicates per treatment/temperature/species was 16. These new treatments were run in early Feb 2022 (*Ulva* only as wild collections of *Hypnea* were unavailable), Apr 2022 (when *Hypnea* became available), and Oct 2022 when day length was similar to that of late 2021 (Sep–Nov) when the first experiments were conducted. See supplementary materials Fig. S1 for a graphical illustration of the full experimental design.

Water temperature inside the bins was regulated by aerating and maintaining the same water levels as in the individual jars. Bin temperatures were recorded every 10 min with HOBO TidbiT MX Data loggers (MX2203, Onset Computer Co., Bourne, MA). Natural irradiance within the shaded experimental environment was measured with a spherical LI-COR 4π sensor (LI-COR Biosciences, Lincoln, NE) submerged upwards in a clear plastic, four-quart container placed centrally and adjacent to the water bins. The sensor was connected to a LI-COR LI-1500 light sensor logger that recorded irradiance (μmols photons m^−2^ s^−1^) every minute from before sunrise (05:00) to after sundown (19:30) for the duration of each experimental run.

The irradiance saturation index, E_k_, describes the point at which E_par_ is optimal and is no longer limiting the individual’s ability to run electron transport at maximum capacity. This parameter alone is informative but to derive more ecologically relevant information, we put E_k_ values into the context of minutes per day each individual experiences E_par_ ≥ E_k_ (termed H_sat_) during the nine-day experiment. We further normalized the total daily H_sat_ relative to the day length (defined as total minutes when E_par_ ≥ 1 μmol of photons m^−2^ s^−1^). See supplementary materials for details on rel-H_sat_ results. As the last step, we developed a diurnal saturated photosynthetic index (DSPI) that incorporates H_sat_ and P_max_.

### Data management and statistical analyses

Photosynthesis data from WinControl were first cleaned of extraneous information and formatted in Python 3 script using PyCharm (v 2021.2.4 Community Edition). The output was imported into R Statistical Software (v2021.09.0 R Core Team, 2012) and used the fitWebb model^58^ in the Phytotools package^59^ to calculate alpha (α) and E_k_. This irradiance-normalized PE model was used to avoid the inherent irradiance dependency of the quantum yield measurements^59^, which are commonly used to calculate ETR. We calculated photosynthesis max (P_max_) via the simple equation P_max_ = α * E_k_ following Silsbe and Kromkamp (2012)^59^. We also calculated relative ETR_max_ (absorption factors for each species were not determined), choosing the highest value during the RLC that satisfied the condition Φ_PSII_ > 0.1 (PSII = photosystem II), following Beer and Axelsson (2004)^66^^60^ protocol for reliable evolved O_2_ to ETR ratios. We compared statistical model fit between P_max_ and rETR_max_ and found P_max_ coefficients of determination were higher for *Hypnea* whereas they were the same for *Ulva*. Thus, we used P_max_ for analysis because it does not violate statistical assumptions of independence and it was a better fit for our statistical models (see supplementary materials for further details). Day 9 values for both P_max_ and E_k_ were used for analyses.

Additional R scripts were written to calculate the time (in minutes) each individual experienced saturating irradiance (H_sat_) during each day of the experiment^49^. For this calculation the 9-day experiment irradiance/E_k_ comparisons were separated into three periods: P1 = days 1–3, P2 = days 4–5, P3 = days 6–9, where E_k_ values from D1 were used with irradiance in P1 to calculate daily H_sat_, D5 E_k_ was used for P2, and D9 E_k_ for P3. A mean was then calculated from these three periods and was used for analysis. Total potential irradiance was shorter for D1 and D9 because the experiments began at 08:00 on D1 and concluded by 12:00 on D9. For the experiment runs in October 2022, D5, RLCs were not run for 48 *Hypnea* (T0) replicates and the comparisons were modified as follows: D1 E_k_ with irradiance on days 1–4 and D9 E_k_ with days 5–9 irradiance. In all cases, irradiance began at the time of the individual’s D1 RLC and ended at the time of its final RLC. All *Ulva* replicates in T2.5 were removed from analysis after many of these experienced high tissue losses associated with lunar cycle effects of reproduction. Two *Hypnea* individuals lacked pigmentation on D9 and were unable to sustain an F_t_ sufficient to run an RLC. These individuals were also removed from the analyses.

To account for differing amounts of daylight throughout the months-long experimental period, day length was calculated from a condensed LI-COR dataset as time E_par_ was ≥ 1 μmol photons m^−2^ s^−1^. This large dataset was condensed first by taking a 10-min moving average and then by excluding all but the values at times :00, :10, :20, :30, :40, and :50 from the analysis. Then, the mean number of minutes in saturated photosynthesis, H_sat_, for each individual each day, was divided by the mean day length for each day. These values were averaged to arrive at a proportion (%) of diurnal daylight each spent in saturating irradiance (rel-H_sat_; see supplementary materials).

### A new photosynthesis approach

A diurnal saturated photosynthesis index (DSPI) was developed to represent the total daily photosynthetic production based on the total time spent in saturating irradiances and assuming each replicate plant ran photosynthesis at a maximum rate the entire time in H_sat_. The DSPI was calculated for each individual using a simplified equation (per Zimmerman et al. 1994 ^49^): P_max_ * H_sat_. It is important to note that the data for this approach are derived from PAM measurements that do not calculate diurnal losses from respiration or other processes not related to irradiance. The units of measurement for P_max_ were first converted from seconds to minutes to match H_sat_ units. The equation was used to calculate daily values for each period as previously described, that were then averaged for a final DSPI expressed in mol m^−2^.

Relationships between treatment and temperature (fixed effects) and photosynthesis parameters (P_max_, E_k_) and iterations of these (H_sat_, rel-H_sat_, DSPI), and growth were analyzed using linear mixed-effects model (LMM) packages lme4 and lmerTest in R (v1.1–31, ^61^). Fixed effects included treatment and temperature for all analyses and for both species. Random effects included: (a) plant ID (to account for natural plant variability), (b) run (potential irradiance changes over time), (c) RLC order (replicate measurement order), and (d) lunar phase (affects *Ulva lactuca* reproduction and growth^62, 63^). All or a subset of these random effects were used for each analysis to achieve the best model fit and avoid issues of singularity. Plant ID was used in all analyses. Residual plots were visually examined to ensure no deviations from normality or homoscedasticity occurred. The Performance package in R (v 0.10.2 ^64^) was used to visualize important model assumptions, i.e., collinearity, influential observations, linearity of residuals, etc. Marginal (fixed effects only; R^2^m) and conditional (fixed + random effects; R^2^m) coefficients of determination were used to determine goodness of model fit. P-values were obtained via likelihood ratio tests that compared the full model with a null model that lacked the effect being tested. Lastly, we ran basic linear regressions with growth as the dependent variable and P_max_, E_k_, H_sat_, rel-H_sat_, and DSPI as predictors to gauge importance as predictors. From this, DSPI as a predictor of growth was modeled with LMM as above using treatment as the random effect. Graphs were plotted using ggplot2 graphic package (v3.3.6, ^65^).

### Algal growth

Growth was calculated using the equation

$$\left(\frac{{w}_{f}- {w}_{i}}{{w}_{i}}\right)*100$$

where f = final (D9) and i = initial wet weight (w; D1). This equation was used to eliminate the need for assumptions of steady or exponential growth because such assumptions have not been well tested or documented in *Ulva* or *Hypnea*^66^. The parasite *Hypneocolax stellaris* was occasionally found on *Hypnea* and was removed and biomass was quantified and subtracted from the plant weight. Details in supplementary materials.

### Research compliance statement

All experiments were conducted in accordance with University of Hawai‘i at Mānoa (UHM) policies and regulations and all relevant lab personnel were trained and in compliance with (UHM) lab safety requirements.

### Specimen collection statement

The State of Hawai‘i does not restrict nor require permission for the collection of algae except for within marine protected areas (MPAs) or if collecting select taxa in the genus *Gracilaria*. Accordingly, all *Ulva* and *Hypnea* specimens were collected outside of the MPAs and are therefore in compliance with State requirements.

### Supplementary Information

Supplementary Information.

## Data Availability

All supporting data are available at author’s (ARD) github repository with username: angelardhawaii. https://github.com/angelardhawaii?tab=repositories.
