# Supplementary material for: Divergent responses of native and invasive macroalgae to submarine groundwater discharge
Source: Sci Rep. 2023 Aug 26;13:13984. doi: 10.1038/s41598-023-40854-7 (PMC10460400; doi:10.1038/s41598-023-40854-7)
Supplement: Supplementary file 1 — Supplementary Information. [file 41598_2023_40854_MOESM1_ESM.docx]

Divergent responses of native and invasive macroalgae to submarine groundwater discharge

*Angela Richards Donà^1^, Celia M. Smith^1^, Leah Bremer^2, 3^,

^1^ School of Life Sciences, University of Hawai‘i at Mānoa, Honolulu, HI, USA

^2^ University of Hawai‘i Economic Research Organization, University of Hawai‘i at Mānoa, Honolulu, HI USA

^3^ Water Resources Research Center, University of Hawai‘i at Mānoa, Honolulu, HI USA

**Supplementary materials**

*Illustration of experimental design*

Figure S1 details the full procedures for (A) The main experiment comprising four treatments with eight iterations to total n=16 replicates. (B) This section outlines the procedures employed to avoid or minimize bin and/or light effects, which included moving jars within bins to new bins and moving jars from front row to back and vice versa. (C) Additional experiments followed the same procedures as the main experiments but with some differences due to availability of macroalgal specimens. All individuals for all experimental runs were in jars within bins under a homogeneously shaded PVC structure in natural light. Bins were heated (or not) according to the treatment and temperatures were adjusted within bins as required by new contents. Bin content changes occurred every two days concurrent with water changes and nutrient inoculations. For full description see methods and materials.


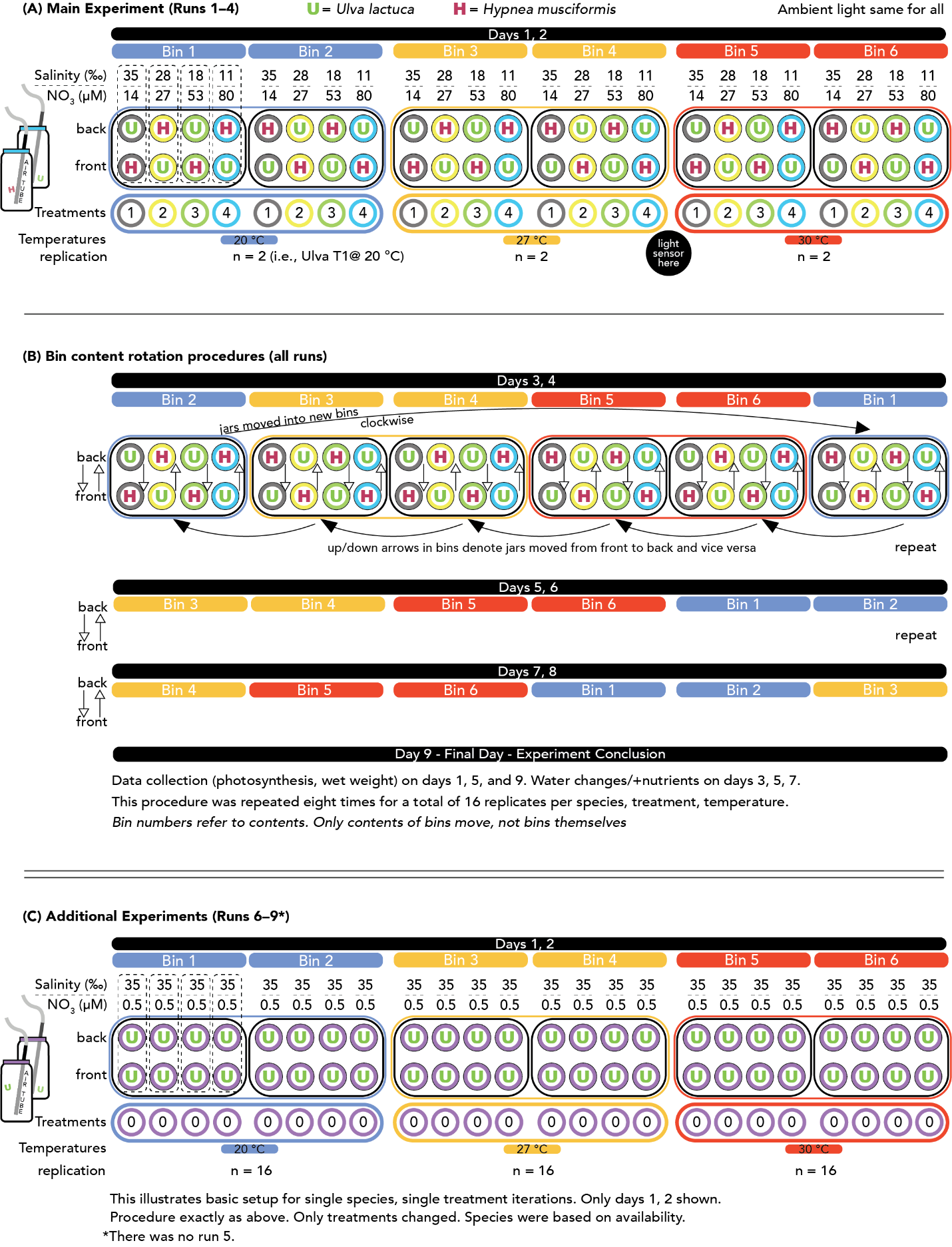


Figure S1

*Results for Relative daily saturation time (rel-H_sat_)*

The mean rel-H_sat_ for *Ulva* was highest for T0 (81.1 %) and declined to 56.0% in T4, representing conditions most similar to those at groundwater seeps (Fig. S2A). Treatment conditions affected rel-H_sat_ (𝜒^2^ (4) = 92.5, p <0.001), decreasing it by a maximum of 26.2% (± 6.3 std. err.), whereas temperature had no significant effect (𝜒^2^ (2) = 2.6, p = 0.273).


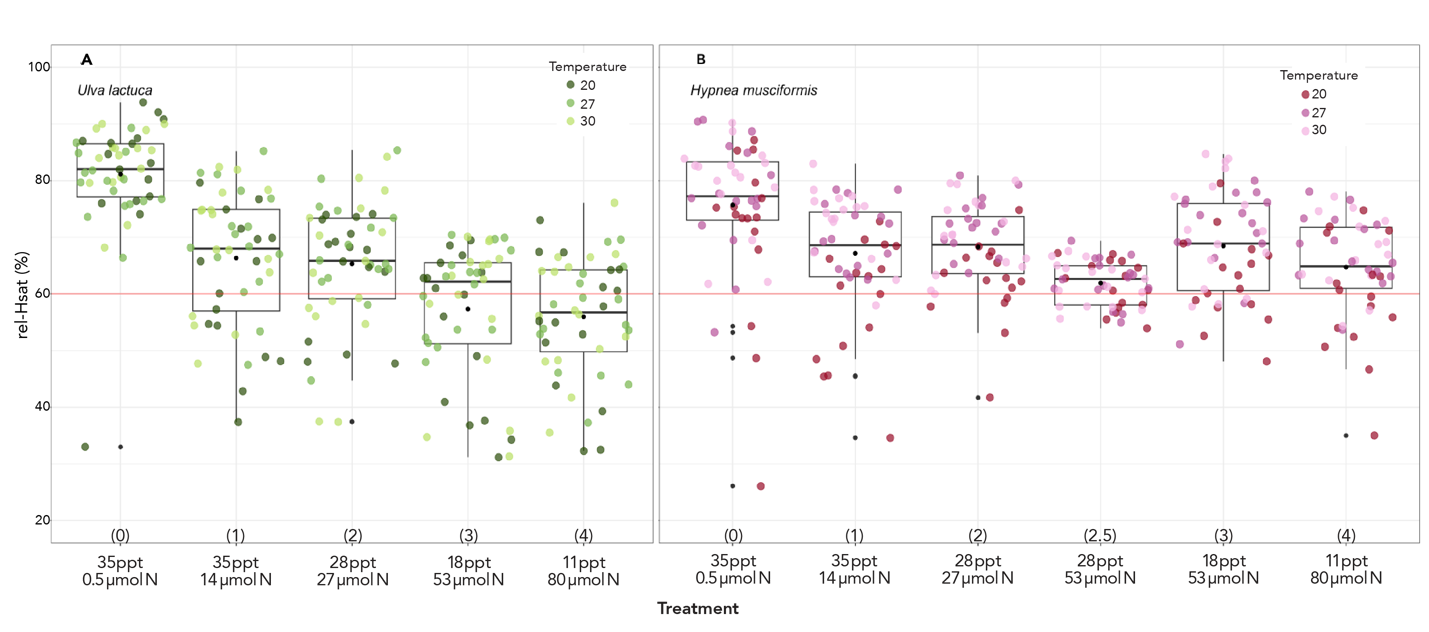
In *Hypnea* both treatment and temperature affected rel-H_sat_ (Fig. S2B). The maximum decrease in rel-H_sat_ (𝜒^2^ (5) = 26.0, p <0.001) relative to baseline T0 was 13.8% (± 3.4 std. err.; T2.5). Temperature increased rel-H_sat_ (𝜒^2^ (2) = 18.3, p <0.001) by 6.6% (±1.8 std. err.) at 27 °C and 7.2% (±1.7 std. err.) at 30 °C, relative to the 20 °C individuals.

Figure S2

Table S1. Statistical output for linear mixed-effects model (LMM) fit for rel-H_sat_ providing marginal and conditional R^2^ values. P-values, chi squared (𝜒^2^ ), and degrees of freedom (df) obtained from likelihood ratio tests for effects of treatment and temperature. Data are divided by species. Total number of observations for each analysis: *Ulva* = 240, *Hypnea* = 286. Random effects in parentheses below R^2^c (a = plant ID, b = run, c = RLC order).

|  | *Ulva* | | | | | | *Hypnea* | | | | | |
| --- | --- | --- | --- | --- | --- | --- | --- | --- | --- | --- | --- | --- |
|  | Likelihood ratio tests | | | | LMM  fit | | Likelihood ratio tests | | | | LMM  fit | |
|  | Treatment | | Temperature | |  |  | Treatment | | Temperature | |  |  |
|  | p | 𝜒^2^ (df) | p | 𝜒^2^ (df) | R^2^m | R^2^c | p | 𝜒^2^ (df) | p | 𝜒^2^(df) | R^2^m | R^2^c |
| rel-H_sat_ | <0.001 | 92.5(4) | 0.273 | 2.6(2) | 0.41 | 0.83  (abc) | <0.001 | 26.0(5) | <0.001 | 18.3(2) | 0.28 | 0.70  (abc) |

Table S2. Mean value for each species for rel-H_sat_ (%) by treatment.

| Treatment | 35‰/0.5μmol N  (T0) | 35‰/14μmol N  (T1) | 28‰/27μmol N  (T2) | 28‰/53μmol N  (T2.5) | 18‰/53μmol N  (T3) | 11‰/80μmol N  (T4) |
| --- | --- | --- | --- | --- | --- | --- |
| *Ulva* | 507 | 404 | 398 | NA | 344 | 333 |
| *Hypnea* | 469 | 469 | 421 | 410 | 420 | 394 |

*Temperature Results*

Table S3. Mean values for photosynthetic dependent variables by temperature with direction of and % change (Δ) from 20 to 27 °C (statistically same as 30 °C) for significant effects only, denoted (*). ^Ŧ^rel-H_sat_ (already a percentage) was simply subtracted.

|  | P_max_ | | E_k_ | | H_sat_ | | rel-H_sat_ | | DSPI | | Growth | |
| --- | --- | --- | --- | --- | --- | --- | --- | --- | --- | --- | --- | --- |
| (°C) | Ul | Hm* | Ul* | Hm* | Ul | Hm* | Ul | Hm* | Ul | Hm* | Ul | Hm |
| 20 | 59.7 | 62.0 | 83.6 | 97.9 | 384 | 388 | 63.0 | 62.4 | 4.24 | 4.56 | 34.8 | 28.2 |
| 27 | 54.4 | 44.4 | 72.9 | 61.5 | 407 | 439 | 66.5 | 70.2 | 4.32 | 4.26 | 34.3 | 23.6 |
| 30 | 55.8 | 41.4 | 72.1 | 58.5 | 400 | 438 | 66.1 | 70.7 | 4.31 | 4.16 | 32.0 | 23.4 |
| Δ | NA | -28% | -13% | -37% | NA | 13% | NA | 8%^Ŧ^ | NA | -7% | NA | NA |

*Photosynthesis measurement considerations*

Ecophysiological investigations involving measurements of photosynthesis are becoming more common with the innovation and use of Pulse-Amplitude-Modulation (PAM) fluorometry that quickly and conveniently measure *in vivo* fluorescence of a photosynthetic sample^1^. Generally, they are used to estimate the efficiency of photosystem II (PSII) photochemistry via rapid light curves (RLCs) that calculate electron transport rates (ETR) from the stepwise fluorescence measurements at increasing irradiance. This simple yet powerful instrument permits researchers to probe impacts of abiotic factors, i.e., irradiance, temperature, and salinity, on algal photosynthesis. It is widely used and considered a reliable proxy for photosynthetic efficiency without the need for lengthy incubations to measure gross O_2_ evolution^2^. An important caveat for its use requires recognition of the instrument’s limited ability to accurately capture the O^2^ to ETR ratio during high irradiance measurements. Beer & Axelsson^2^ showed that the O^2^: ETR relationship in *Ulva lactuca* (among others) breaks down when quantum yield of PSII (Φ_PSII_) is less than 0.1. This is an important consideration when communicating results, which are commonly reported as maximum electron transport rates (ETR_max_). Electron transport is calculated at each step of the RLC as ETR = Φ_PSII_ * irradiance (E_par_) * the specific absorption factor (AF) for the sample. Relative ETR_max_ (rETR_max_; Φ_PSII_ * E_par_) is used when the exact absorption coefficient for the measured specimen(s) is unknown^2,^ ^3^. Furthermore, the E_par_-dependency of the calculation for rETR (or ETR) is often overlooked yet violates an important statistical requirement. Silsbe and Kromkamp (2012)^4^ provide a method (and an R package “Phytotools”) to directly model the E_par_ -dependency of Φ_PSII_ for robust analysis of photosynthetic parameters, 𝛼 (initial slope of light curve) and light saturation index (E_k_). Taking these important stipulations into consideration for this study, we used 𝛼 and E_k_ values calculated from fitting an E_par_-normalized model, fitWebb^5^, the most robust model tested by Silsbe and Kromkamp (2012)^4^. We further calculated an alternative rETR_max_ (P_max_) from the fitWebb output as described in the text.

*Parasitic rhodophyte on* Hypnea musciformis *axes*

The parasite *Hypneocolax stellaris* was occasionally found on *Hypnea* and prompted a count from all photos to estimate the total biomass contributed by the parasite across all experimental runs. To determine mean biomass weights (g) for small (~ ≤1 mm^3^) or large (~1–2mm^3^) sized classes, parasites were excised from eight separately collected *Hypnea* plants and collectively weighed in either of the two size classes. These values were divided by the total number of excised parasites per class (92 small and 29 large) to calculate a mean size for small (0.00075 g) and large parasites (0.0019 g); this value was subtracted from *Hypnea* tissue when the parasites were too difficult to remove and re-weigh the plant. Photos where parasites were identified were counted at least twice and numbers of parasites in D1 photos were subtracted from those of D9 photos. D9 weight was adjusted by multiplying the number of parasites found per size class by the mean value for that size. For replicates that had parasites on D1 but not on D9, the adjustment was applied to D1 weight. Although we removed and quantified the weight of the parasites, we didn’t directly test the effect they had on *H. musciformis* growth. This is a consideration that should be evaluated when working with this species in the future.

**Figure legends**

Figure S1. Illustration of experimental design. (Top) Main experiment basic setup for days 1, 2. This produced n=16 replicates after eight iterations of the same experiment including four treatments (T1–T4), three temperatures (~20, 27, and 30 °C), and two species, *Ulva lactuca* (U) and *Hypnea musciformis* (H). (Middle) main experiment days 3, 4 showing procedure for rotating bin contents clockwise into new bins and jars from front to back within new bins. Bins did not move, temperatures in bins were adjusted. This occurred every two days until conclusion on day 9. (Bottom) Additional experiment basic setup for days 1, 2 shows a single treatment/single species iteration. Replication n=16. Bin rotation, data collection, water changes, and nutrient inoculations occurred as in main experiment. Runs 1–4 ran continuously from Sept to Nov 2021. Runs 6–9 occurred in Feb, April, and October 2022. There was no run 5.

Figure S2. Rel-H_sat_ (%) plotted by treatment for (A) *Ulva* and (B) *Hypnea*. Black dots denote means for each treatment. Treatment description and number (in parentheses) on x-axis. Shade of colored dot represents temperature. Red lines mark 60%, an arbitrary value for comparison.

**References**

1. Schreiber, U. Pulse-Amplitude-Modulation (PAM) Fluorometry and Saturation Pulse Method: An Overview. in *Chlorophyll a Fluorescence: A Signature of Photosynthesis* (eds. Papageorgiou, G. & Govindjee) 279–319 (Springer, 2004). doi:10.1300/j301v01n03_06.

2. Beer, S. & Axelsson, L. Limitations in the use of PAM fluorometry for measuring photosynthetic rates of macroalgae at high irradiances. *Eur J Phycol* **39**, 1–7 (2004).

3. Ralph, P. J., Gademann, R., Larkum, A. W. D. & Kühl, M. Spatial heterogeneity in active chlorophyll fluorescence and PSII activity of coral tissues. *Mar Biol* **141**, 639–646 (2002).

4. Silsbe, G. M. & Kromkamp, J. C. Modeling the irradiance dependency of the quantum efficiency of photosynthesis. *Limnol Oceanogr Methods* **10**, 645–652 (2012).

5. Webb, W. L., Newton, M., Starr, D. & Url, S. Carbon Dioxide Exchange of Alnus rubra . A Mathematical Model. *Oecologia* **17**, 281–291 (1974).
